# Expression of an Innate Immune Element (Mouse Hepcidin-1) in Baculovirus Expression System and the Comparison of Its Function with Synthetic Human Hepcidin-25

**Published:** 2011

**Authors:** Yaghoub Yazdani, Hamid Sadeghi, Mohammad Alimohammadian, Alireza Andalib, Fatemeh Moazen, Abbas Rezaei

**Affiliations:** a *Department of Molecular Medicine, Faculty of Advanced Medical Science Technologies, Golestan University of Medical Sciences, Gorgan, Iran.*; b *Department of Immunology, School of Medicine, Isfahan University of Medical Sciences, Isfahan, Iran.*; c *Department of Biotechnology, Isfahan Pharmaceutical Science Research Center, School of Pharmacy, Isfahan University of Medical Sciences, Isfahan, Iran.*; d *Department of Immunology, Pasteur Institute of Iran, Tehran, Iran.*

**Keywords:** Hepcidin, Baculovirus expression system, Functional study, J774A.1

## Abstract

Hepcidin is an innate immune element which decreases the iron absorption from diet and iron releasing from macrophage cell**. **In contrast to the chemical iron chelators, there has been limited effort applied to the specific use of hepcidin as a new drug for decreasing the iron overload.

Hepcidin is produced in different biological systems. For instance, *E-coli *is used for human hepcidin expression, however, post-translational modification is impaired. We have used a simple baculovirus expression system (BES) to improve the hepcidin folding and activity. Hepcidin Messenger Ribonucleic acid (mRNA) was isolated from mouse liver cells and its complementary Deoxyribonucleic acid (cDNA) was produced and amplified. PFastBac HTB vector was used for recombinant bacmid production. Recombinant baculovirus was produced using SF-9 cell line. The mouse hepcidin-1 protein was expressed in a large quantity and functional tests were performed for this recombinant peptide. The yield of hepcidin in BES was 20 μg/mL and anti-histidine (anti-His) tag antibody was used for the confirmation of hepcidin on western blot nitrocellulose paper. Functional tests showed that mouse hepcidin accumulates iron in the macrophage cell line J774A.1 up to 63%. In addition, our data showed that the mouse hepcidin-1 has less toxicity compared to the synthetic human hepcidin-25 (p = 0.000).

## Introduction

Hepcidin was originally isolated from the human serum and urine (1, 2). Hepcidin is predominantly found in liver cell and weekly express in stomach, intestine, colon, heart, thymus and alveolar macrophages cells (3, 4). Sequential analysis of protein has shown that hepcidin is a cysteine rich peptide with several disulphide bonds (5). It is conserved among species from fishes to mammals (2). Humans have only one copy of the hepcidin gene, however, mice have two copies named hepcidin-1 and hepcidin-2 (3). Mouse hepcidin gene consists of 3 exons and 2 introns which is located on chromosome-7 (3). Reports show that mouse hepcidin-1 (but not hepcidin-2) involve in iron regulation, iron storage and hemoglobin level (6, 7). Hepcidin causes internalization and degradation of iron exporter ferroportin, which is present on the cell surface of macrophage and enterocyte cells. Thus, hepcidin inhibits the release of iron by macrophages and iron uptake by enterocytes (8).

Antimicrobial activity against several microorganisms were also pointed in hepcidin derived from different species (9, 10). Moreover, hepcidin as an iron inhibitor represents an important class of anti-tumor agents since iron is an essential trace element that is vital for DNA synthesis (11).

Iron overload is the dangerous side effect of blood transfusion that occurs in hemoglobinopathies, such as thalassemia (12, 13). Deferiprone and deferoxamine are routinely used for removal excess iron (14). In contrast to the chemical iron chelators, there has been limited effort applied to the specific use of hepcidin for decreasing iron overload in serum.

In spite of the reports describing the effect of hepcidin on iron regulation in the literature, there is little information about mouse hepcidin-1 effect on iron accumulation and also cell viability on macrophage cells, as important cells that participate in iron metabolism. 

Hepcidin can be extracted through purification from plasma and urine, but the yield of purified crude hepcidin in this way is low (2). The second way is synthesis of hepcidin. Preparation pathway of functional synthetic hepcidin is very difficult and refolding procedures are required (15). Alternatively, hepcidin can be produced in bacterial expression system. The advantage of this expression system is its low cost and high productivity, but its post-translational modifications can be impaired in comparison with the expression in mammalian cells (16).

We tried to use a simple biological expression system that might have more similarity to mammalian cells. Therefore, we hypothesis that the baculovirus expression system (BES) would be efficient for this purpose (17, 18). The other advantage of this system is the lack of contamination with bacterial component such as LPS and hence, applying this protein would not activate immune responses (19).

We decided to use BES for cloning and expression of mouse hepcidin-1 peptide. In this study, isolation and cloning of the mouse mRNA encoding hepcidin protein was carried out and its production in baculovirus expression system was considered. For functional assessment, we compared mouse hepcidin-1 and synthetic human hepcidin-25 effect on iron concentration and cell viability in J774A.1 cells line. To the best of our knowledge, the present study is the first work that considers all the above aspects for expression and functional assessment of mouse hepcidin-1 in the baculovirus expression system.

## Experimental

*Cloning and production of recombinant baculovirus*

The liver of sacrificed male C57/Bl6 mice was isolated and its total mRNA was extracted from liver cells using RNeasy Mini Kit (Qiagen, Germany). Then, the hepcidin cDNA was amplified utilizing Qiagen one-step RT-PCR Kit. The primer sequences were as follows: forward primer (*BamHI*), 5-GGATCCATGGCACTCAG CACTCGGAC-3 and reverse primer (*XbaI*), 5-TCTAGAGGCTCT A GGCTATGTTTTGC-3. Patterns of digestion with ALU I enzyme was used for the preliminary assessment of PCR product.

The purified PCR product was ligated into the “PTZ57R/T” vector. One of the obtained recombinant plasmids, was then sequenced (Macrogen, Korea). Subsequently, this plasmid was digested with BamHI and XbaI restriction enzymes and then the insertion was subcloned into the “PFastBac HTB” plasmid (Invitrogen, USA). The insertion caused the addition of a hexa histidine sequence into the 5´ end of the hepcidin cDNA. The fidelity of the insertion in PFastBac B vector was confirmed by PCR analysis using polyhedrin primer and reverse primer of hepcidin cDNA and digestion with NcoI enzyme. Four selected recombinant plasmids were then sequenced (Macrogen, Korea) to verify the orientation and proper framing of insertion.

The selected recombinant vector was used for the production of recombinant bacmid. Afterward, the bacmids were isolated utilizing midiprep kit (Invitrogen, USA) from the selected colonies. 

Insect SF9 cell line (Invitrogen, USA) was used for the production of recombinant baculovirus. Before the transfection, cells were grown (27°C) using “SF-900 II SFM” cell culture medium (Invitrogen, USA) supplemented with 1x penicillin/streptomycin/neomycin(Gibco BRL, USA) in order to adopt them to this growth condition. According to manufacturer’s instruction, the transfection of these cells occurred using cellfectin reagent. After the cells showed signs of late stage of infection, the medium containing the baculovirus was collected and the amount of it was calculated.

*Expression and analysis of mouse hepcidin-1*

For hepcidin expression, multiplicity of infections (MOIs) 5, 10 and 20 were utilized in monolayer SF-9 cell (20). Infected SF-9 cells were harvested 48, 72 and 96 h post-infection for hepcidin expression analysis. Expression of the recombinant mouse hepcidin-1 in the SF-9 cell was examined by sodium dodecyl sulfate polyacrylamide gel electrophoresis (SDS-PAGE) and Western blot analysis. Samples were electrophoresed in 15% SDS PAGE gels. After the electrophoresis, the proteins were transferred to a nitrocellulose membrane (Sartorius AG-USA) at 400 mA for 45 min using an electroblot system (Bio-Rad, USA). Membranes were then washed extensively with PBS buffer (0.15M, pH 7.4) and blocked with 3% of skim milk (Sigma, USA). The membranes were shacked for 1.5 h at room temperature with anti His-tag antibody (Calbiochem, USA) at final concentration of 4 μg/mL in PBS buffer, containing 2% bovine serum albumin (BSA) (Sigma, USA). After stringent washing using PBS-Tween-BSA solution (0.05% Tween, 2% BSA), the membranes were incubated with 1 : 1000 HRP conjugated sheep anti mouse (Sigma, USA) for 1.5 h. After washing the membranes, hepcidin was detected using DAB as the substrate for HRP and appearance of brown colored protein bands on the membrane (Sigma, USA).

*The hepcidin effect on iron concentration*

The His-tag bounded at recombinant mouse hepcidin-1 was cleaved with enterokinase enzyme according to manufacturer’s instruction (Promega, USA). Human hepcidin refolded using refolded buffer (20 mM Tris, pH 7.4, 150 mM NaCl, 100 mM KCl, 5 mM GSH and 0.5 mM GSSG) at final concentration of 100 μg/mL (incubated 24 h in 4°C). J774A.1 cell line (Pasteur Institute, Iran), was utilized for evaluating the hepcidin effect on iron export. The cells were cultured in DMEM medium (Sigma, USA) supplemented with 1x penicillin, streptomycin, neomycin (Invitrogen, USA) and 10% fetal bovine serum (Sigma, USA).

The cells were seeded into a 6-well plate with 1 × 10^6^ cells/well in 2 mL DMEM medium and incubated for 24 h simultaneously with 4 or 8 μg of hepcidin. In another groups, synthetic human hepcidin-25 (PRIMM, Italy) and BSA (dissolved in refolding buffer) were used with the same concentration. After incubation, cells were washed three times with PBS (0.15 M, pH = 7.5) and then lysed in 2% SDS. Eventually, total proteins were precipitate with 20% trichloroacetic acid and free iron was measured in supernatant using atomic absorption tool (Perkin Elmer, 2380, USA).

*The hepcidin effect on cell viability*

Cell viability for hepcidin was examined using the MTT (3-(4, 5-dimethylthiazol-2-yl)-2, 5 diphenyl tetrazolium) proliferation assay (21).

The J774A.1 cell line was seeded into a 24-well plate at a density of 4 × 10^4^ cells/well in 1 mL DMEM medium. Recombinant mouse hepcidin-1 was added to the wells with the concentrations of 2, 3, 4, 5, 6, 7 and 8 μg/well. Synthetic human hepcidin-25 and BSA were also used with the same concentration in the other groups. Then, plates were incubated for 24 h at 37°C. Briefly, MTT was added to wells at the final concentration of 0.5 mg/mL and the plates were incubated for 4 h at 37°C. Thereafter, medium was removed and the cells were lysed in DMSO. Subsequently the conversion of MTT to formazan, corresponding to cell viability, was assessed by ELISA reader at 540 nm.

## Results and Discussion

*Expression of mouse hepcidin-1*

**Figure 1:**
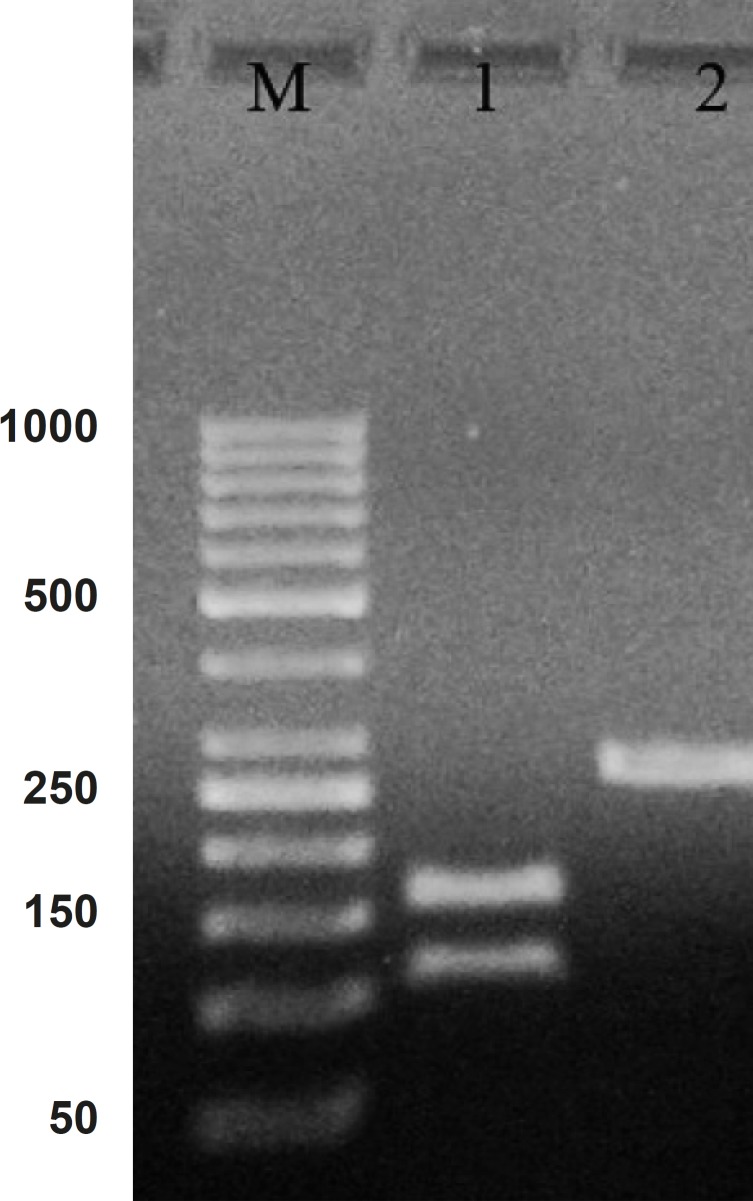
PCR product of hepcidin cDNA in 1% agarose gel electrophoresis. Lane M is the molecular marker (GeneRuler™ 50 bp DNA Ladder). Lane 1 is the effect of ALU-1 restriction enzyme on PCR product confirming it as a hepcidin cDNA. Lane 2 is PCR product with 260 bp weight expected for hepcidin cDNA

Hepcidin cDNA was amplified using specific primers (mentioned in materials and methods). After the gel electrophoresis, the amplified cDNA was appeared as a clear band at 260 bp (Figure 1). 

Comparing the cDNA pattern with the patterns present at NCBI information, confirmed that the amplified segment would be hepcidin cDNA. The Lane 2 in Figure 1 indicate a digested pattern of amplified cDNA after the treatment with *ALU*-I restriction enzyme. Nucleotide sequential determination of hepcidin cDNA indicated that the sequence pattern had only one restriction site for the *ALU*-I enzyme at the position of 112. As a result of digestion, one segment with 112 bp and one with 157 bp were displayed in the pattern of gel electrophoresis.

**Figure 2:**
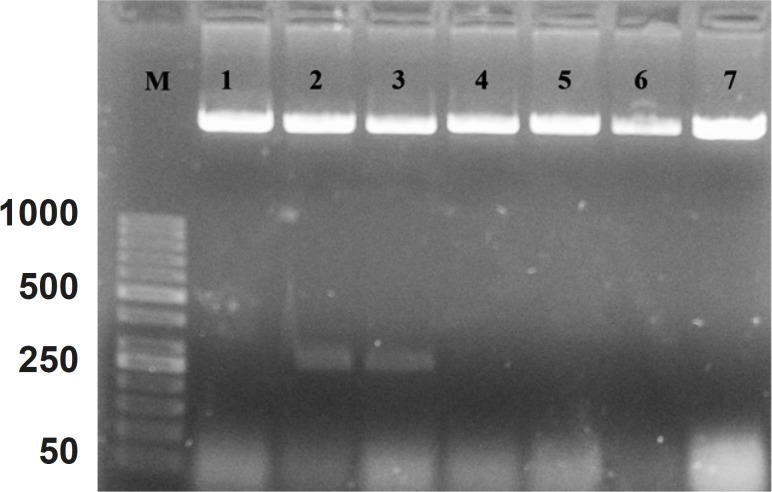
Patterns of “PFastBac HTB” digestion with NcoI enzyme on the obtained recombinant plasmids

**Figure 3:**
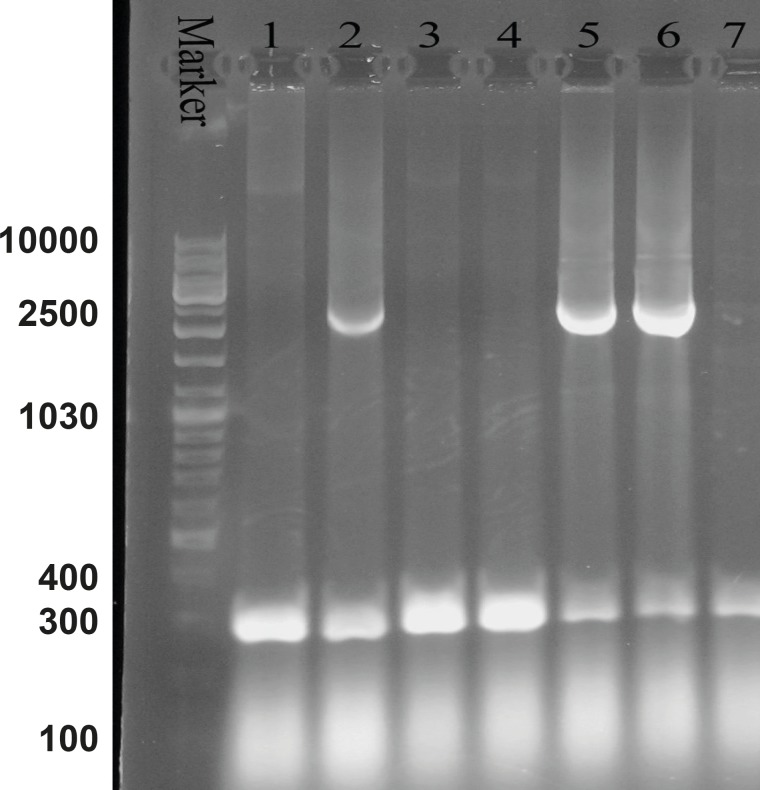
Agarose gel electrophoresis of the recombinant bacmid. Lane M is the molecular marker (GeneRuler™ DNA Ladder Mix). Lanes 1, 3, 4 and 7 show the 300 bp bands which confirm that the homologous recombination for bacmids has not occurred. Lanes 2, 5 and 6 illustrate the 2680 bp amplified bands, confirming the correct homologous recombination

The cDNA was inserted into “PFastBac HTB” and recombinant vector was produced. The DNASIS software (Hitachi software engineering, Japan) was applied to look for the pattern of restriction sites on the recombinant vector. Then, the *NcoI *enzyme was selected for the determination of the inserting orientation in the cloning site. After the analysis, it was found that the recombinant vector had one “restriction site” before “cloning site” on “PFastBac HTB plasmid” and one “restriction site” on forward primer. Figure 2 shows the orientation of hepcidin cDNA in cloning site of “PFastBac HTB vector”. The lanes 2 and 3 indicate that the target gene is in wrong orientation. The colonies that have wrong orientation were discarded from this step. Those colonies with correct orientation were used for sequencing. Afterward, recombinant bacmid was produced when DH10 cells were transformed by recombinant “PFastBac HTB” vector (Figure 3).

Lane M is the molecular marker (GeneRuler™ 50 bp DNA Ladder). Lanes 1, 4, 5, 6 and 7, show correct orientation of hepcidin cDNA in the cloning site of recombinant bacmid. Lanes 2 and 3 show a band with approximately 260 bp, confirming the wrong orientation of hepcidin cDNA in the cloning site of “PFastBac HTB” vector.

M13 forward and reverse primers were utilized for the evaluation of recombinant bacmid. The Pattern of gel electrophoresis illustrates the occurrence of proper homologous recombination between recombinant vector and bacmid genome (Figure 3: Lanes 2, 5 and 6). Subsequently, SF-9 cells were transfected with the recombinant bacmid and the baculoviruses were successfully produced as a result of this transfection. Ninety-nine h post-transfection of the cells, the baculoviruses were collected from the lysed cells. After the baculoviruses got amplified in the SF-9 cells in sufficient amount, the MOIs 5, 10 and 20 were used for protein expression assessment.

**Figure 4:**
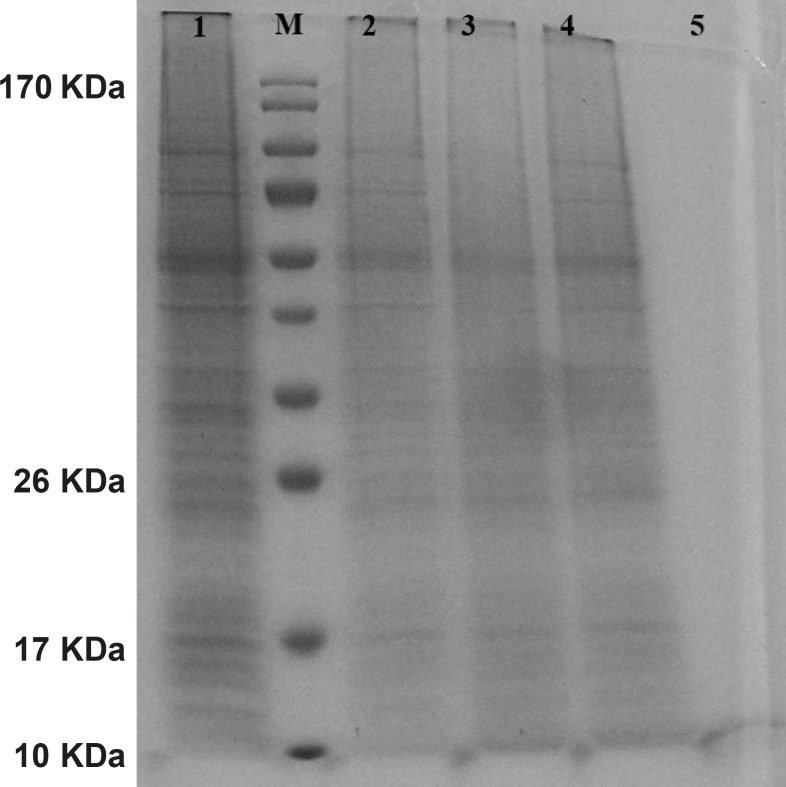
Protein pattern of cells transfected with mouse hepcidin-1 electrophoresed in 15% SDS-Polyacrylamide Gel. Lane M is the molecular marker (Pageruler™ Prestained Protein Ladder). The lane 1 is the pattern of total protein products isolated from SF-9 cells transfected with natural baculovirus (without hepcidin gene) as negative control. Lanes 2, 3 and 4 are protein products after 24 h from SF-9 cells transfected with recombinant bacmid with MOI.5, 10 and 20 respectively. As it is seen, more expressions are obtained with more MOI. Lane 5 illustrates a pure 10 KDa band of mouse hepcidin-1 after passing the total protein products through nickel columns for the purification process

**Figure 5:**
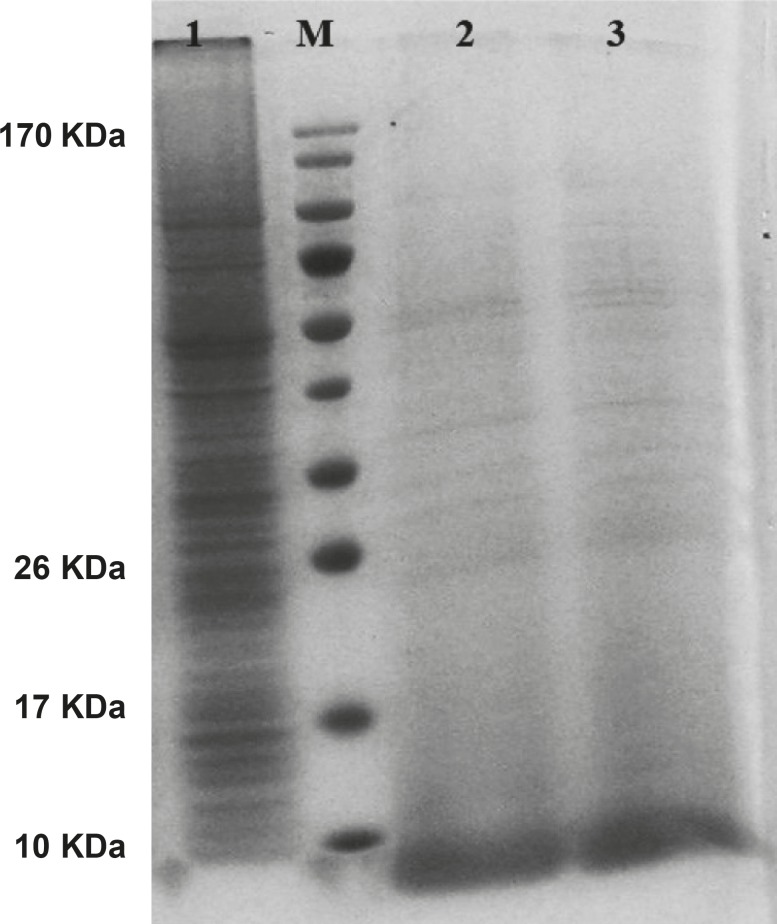
Mouse hepcidin-1 expression in SF-9 cells and the electrophoresis of the proteins in 15% SDS-Polyacrylamide Gel.The lane M is the molecular marker (Pageruler™ Prestained Protein Ladder). The lane 1 is the pattern of total protein products isolated from SF-9 cells transfected with natural baculovirus (without hepcidin gene). The lane 2 is the total of proteins isolated from supernatant of SF-9 cells transfected with recombinant bacmid (with the hepcidin gene) with MOI.20 in 72 h post-incubation time period. Mouse hepcidine-1 protein is approximately 10 KDa. The lane 3 has the same conditions as lane 2 except with the 96 h post-incubation time period

**Figure 6:**
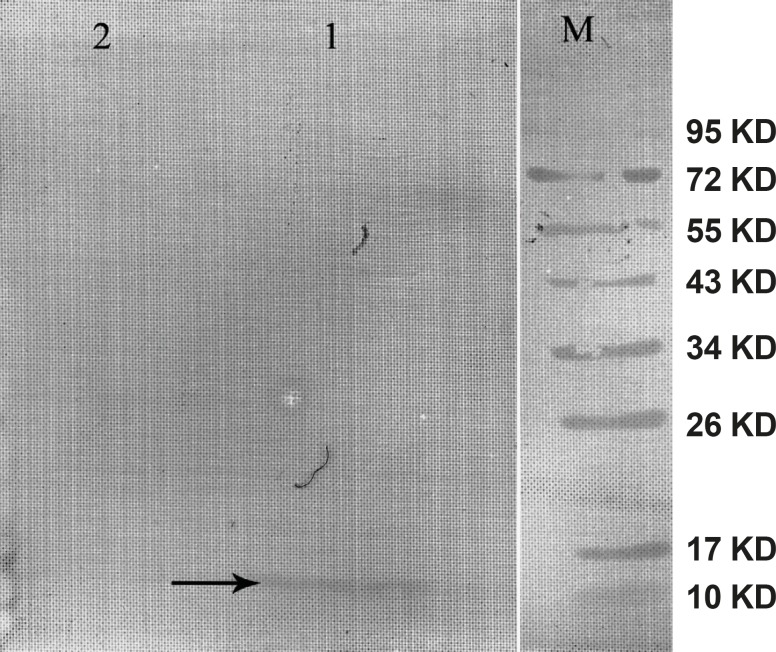
Western blot of the mouse hepcidin-1. The lane M is the molecular marker (Pageruler™ Prestained Protein Ladder). The lane 1 is Anti-His tag antibody binding to 10 KDa protein produced in SF-9 cell transfected with recombinant baculovirus and specifies mouse hepcidin-1. The lane 2 is the negative control. This lane was loaded with total proteins extracted from the SF-9 cells transfected with natural baculovirus (without hepcidin gene

The gene expression of hepcidin in SF-9 cells is illustrated in Figure 4. After 24 h, hepcidin expression in MOIs 5, 10 and 20 resulted in the bands observed in lanes 2, 3 and 4. Moreover, lane 1 shows the expression pattern of all the genes in SF-9 cells which were transfected without using recombinant bacmid (negative control). Hepcidin gene expression bands are shown in lanes 2, 3 and 4 with approximately 10 KDa. However, the lane 1 does not contain the hepcidin band. Hepcidin protein purification was performed by using nickel column and the resulting band is seen in lane 5. The lanes 2 and 3 in Figure 5 show a pattern of all the genes which were expressed in the SF-9 cells transfected with MOI.20. Expression of hepcidin gene is seen in a band of approximately 10 KDa. Our results reveal that the optimum time for the protein expression is 72 h post-infection. Hepcidin expression products were confirmed by western blotting using anti-his tag antibody (Figure 6).

*Comparison of mouse and human hepcidin function*

Functional assessment was performed by purification of hepcidin on nickel column. An amount of 1 × 10^6^ J774A.1 cells were cultured in 12-well plates containing 2 mL of DMEM medium. Then, the cells were incubated with 4 and 8 μg of purified mouse hepcidin-1. Synthetic human hepcidin-25 and bovine serum albumin (BSA) were added with the same concentrations for positive and negative control groups, respectively.

Intra-cellular iron concentration was determined using atomic absorption device 24 h post-incubation. Results of iron concentration are summarized in Table 1. The obtained data showed that in concentration of 4 μg, the effect of the mouse recombinant hepcidin-1 was lower than the synthetic hepcidin-25 on iron concentration (p = 0.016). However, the effect of both types of hepcidin (Table 1) on iron accumulation in the cells was significantly higher than BSA as the control group (p = 0.000).

**Table 1:** The effect of hepcidin on iron accumulation in J774A.1 cell line

**Concentration**	**4 μg**			**8 μg**	
Mouse hepcidin-I	44 ± 1.0			54 ± 1.52	
Human hepcidin-25	47 ± 1.52			56 ± 2.0	
p- value	0.016			0.44	
BSA	29 ± 0.57			33 ± 1.0	
p- value	H	M		H	M
	0.000	0.000	0.000		0.000

The data show the mean ± SD of atomic absorption tool for iron accumulation (ng/10^6 ^cells) in the J774A.1 cells. The comparison of means was performed with one-way ANOVA test and the significant level was p ≤ 0.05. The BSA effect was compared with both human (H) and mouse (M) hepcidin independently.

*Toxicity assay*

Both types of hepcidin with the concentration of 2-8 μg/mL as well as BSA were added separately to the tissue culture wells containing 4 × 10^4^ J774A.1 cells. The viability of the cells was evaluated by MTT assay. The results are summarized in Table 2. The analysis indicates that the viability of the cells with the mouse hepcidin is much higher than synthetic hepcidin with the p- value of 0.000-0.007 for all the concentrations used. In addition, the effect of BSA in cell viability was more similar to the effect of mouse hepcidin-1 than that of human hepcidin-25. In other words, the toxicity of mouse hepcidin is much less than the human synthetic hepcidin-25.

**Table 2:** The effect of hepcidin on cell viability in J774A.1 cell line

**Concentration**	**2 μg**		**3 μg**		**4 μg**		**5 μg**		**6 μg**		**7 μg**		**8 μg**	
Hepcidin-I	2.39 ± 0.015		2.45 ± 0.01		2.5 ± 0.01		2.69 ± 0.015		2.61 ± 0.030		2.49 ± 0.001		2.36 ± 0.052	
Hepcidin-25	2.00 ± 0.1		2.14 ± 0.047		2.24 ± 0.04		2.38 ± 0.076		2.2 ± 0.002		2.15 ± 0.015		2.00 ± 0.01	
p- value	0.000		0.000		0.000		0.001		0.000		0.000		0.007	
BSA	2.41 ± 0.015		2.44 ± 0.01		2.54 ± 0.036		2.64 ± 0.036		2.64 ± 0.072		2.59 ± 0.0917		2.65 ± 0.005	
	H	M	H	M	H	M	H	M	H	M	H	M	H	M
p- value	0.000	0.911	0.000	0.905	0.000	0.339	0.002	0.398	0.000	0.701	0.000	0.000	0.000	0.008

The data is mean ± SD of optical density (OD) resulted from J774A.1 cells treated with hepcidin. After 24 h of incubation time period, the OD obtained from the control group (BSA treated) was compared with both synthetic human hepcidin-25(H) and mouse hepcidin-1 (M). The comparison between the means was performed with one-way ANOVA test and is illustrated by the p- value independently in the row

## Discussion

Hepcidin was purified as a novel peptide from the human blood and was called LEAP-1(1). Park *et al*. (2001) described the same peptide from the liver and due to its origin was named hepcidin (2). Hepcidin is involved in regulation of iron hemostasis (22) and play a role in host defence against microbial invasion (23). In addition, hepcidin gene has been cloned from fish (24, 25), canine (26) and human (2). *Escherichia coli *(27, 28) and *Pichia pastori *were used for their expression (29). Hepcidin characteristics and function are search for the basic science development (30-32) and disease mechanism involvement in clinic (33, 34). In the present study, the effect of mouse hepcidin-1 as a new drug was investigated on iron concentration and cell viability in J774A.1 as macrophage cell line. By designing the specific primers, hepcidin cDNA was amplified from mouse liver mRNA. Consequently, DNA fragment of 260 bp was produced. Previous reports in three independent researches indicated that the mouse hepcidin-1 has 251 nucleotides (3, 35, 36). We added two six bp nucleotide on forward and reveres primers for directional cloning of hepcidin cDNA to the vector. The hepcidin sequence reported by Strausberg (2002) was utilized for the restriction map analysis by DNASIS software. This analysis showed that the target sequence had only one restriction site in the position of 112 for “*ALU*- I” restriction enzyme. This restriction site has been confirmed by Nicolas (35) and Pigeon (3) previously. According to the manufacturer’s instruction, M13 forward and reverse primers are utilized for the assessment recombinant bacmids and a segment with 2430 bp plus the size of insertion segment (251 bp) was seen in positive colonies. Amplified segment in our project indicates that the homologous recombination had occurred (Figure 2: Lanes 2, 5 and 6).

In the present investigation, “*BamHI*” restriction enzyme has cross-reacting with “*XbaI*”. However, previous reports indicate that these enzymes have specific effect on their recognition sites (37). Our experiment shows that two out of seven colonies (35%) (Figure 2) had cross-reacting effect. In other words, the sticky end produced by “*BamHI*”, was bounded to the sticky end produced by “*XbaI*”.

The largest amount of hepcidin was produced in MOI.10 after 24 h post-infection. However, when cells were incubated for 96 h, no significant difference was observed between the MOI.5 and most of the MOIs. In the baculovirus expression system reported by Kim *et al*. (2007), the highest protein expression was in MOI.5. On the other hand, Posse *et al. *(2008) used MOI.5 for optimum conditions, as well (38, 39). The details of our results can be clarified as follows:

The existent baculovirus in MOI.5, used all capacity of insect cell line for the protein expression, therefore, most of MOIs had no effect on this process (40)***.***

The produced hepcidin is a foreign protein in SF-9 cell line. Synthesis of any foreign protein consumes cell sources such as amino acids and may decrease the protein expression in host cell when its concentration increases (41).

Ruan *et al*. (2008) have reported that maximum yield of exogenous protein in SF-9 cell was achieved after 72 h post-infection by recombinant baculovirus (42). Our results show that 72 h would be the optimum condition for production of intact and functional hepcidin. In addition, infection time period was tested for more than 72 h (*i.e*. 96 h) and the hepcidin expression was not different from 72 h. On the western blot analysis, anti-His tag antibody was used for the indirect confirmation of hepcidin expression. Our result revealed that this antibody has not direct correlation with the relative amount of hepcidin expression. For this finding, one possibility is considerable. His-tag epitope is hidden inside the protein, thus, its epitope is not properly in access with the anti-His tag antibody (43).

Researchers have reported that the yield of protein expression was 5-15 μg/mL in baculovirus expression system (44), 55-60 μg/mL in bacterial expression system (45) and 100 μg/mL in fungal expression system (29). The yield of our hepcidin product was 20 μg/mL and this is more appropriate for baculovirus expression system.

According to the reports, if hepcidin binds to ferroportin channel, the channel will become degrade and will inhibit the iron export from the cells (46). Chaston *et al. *indicate that ferroportin levels decreased in hepcidin treated macrophages (47). In addition, reports show that human hepcidin-25 decreases the iron uptake by 50% in Caco-2 cells (intestinal cell line) (48). Our results reveal that iron accumulates up to 63% in J774A.1 cells once treated with mouse hepcidin-1. Human hepcidin in comparison with the mouse hepcidin-1 had more effect with 4 μg dosage on intra-cellular iron concentration (p = 0.016). This achievement can be explained through two possibilities. The first possibility is that the human hepcidin-25 might have more effect on the iron metabolism (15). The other possibility which can explain this achievement, is the fact that recombinant mouse hepcidin might lose some of its activity during the purification and post-purification process (imidazole removement and His-tag cleavage) (49). However, with the dosage of 8 μg, the difference between the two types of hepcidin was not statistically significant (p *= *0.44). These could also be explained by two possibilities. First, this cell line may express distinct levels of ferroportin channel, so the increase in hepcidin dosage treatment have no significant effect on intra-cellular iron concentration (50). Second, cell culture medium has limited the source of iron and the cells have restriction for the iron uptake. Thus, due to the limitation of iron, increasing hepcidin would not effect on iron concentration in the cells.

Physiological and biochemical effects of hepcidin in biological systems are under investigation. Jyh-Yih chen *et al. *stated that fish hepcidin TH2-3 product have cytotoxic effect on HT1080 cell line (51). Our data show that synthetic human hepcidin-25 in comparison with mouse hepcidin-1 or BSA has a higher cytotoxic effect.

In conclusion, during this study, we produced functional mouse hepcidin-1 as a new drug at a high concentration and it was revealed that mouse hepcidin-1 has considerable effect on iron accumulation in macrophage cells and thus, it can decrease iron overload in serum and prevent iron precipitat.
